# Multisystemic Complications and Rapid Decline in Anti‐MDA‐5 Positive Amyopathic Dermatomyositis: A Case Study

**DOI:** 10.1002/ccr3.70261

**Published:** 2025-02-25

**Authors:** Hem Prajapati, Yesha R. Chauhan, Sahaj Y. Patel, Ajay C. Parmar, Tasin Mohammedyakub Shaikhjiwala

**Affiliations:** ^1^ Intern, Government Medical College Vadodara India; ^2^ Intern at Medical College Baroda, SSGH Vadodara India; ^3^ Second Year Resident at Department of General Medicine S.S.G. Hospital & Medical College Baroda Vadodara India; ^4^ Second Year Resident in Internal Medicine at Department of General Medicine S.S.G. Hospital and Medical College Baroda Vadodara India; ^5^ Bukovinian State Medical University Chernivtsi Ukraine

**Keywords:** amyopathic dermatomyositis, dermatomyositis, interstitial, lung diseases

## Abstract

Anti‐MDA‐5 positive amyopathic dermatomyositis (CADM) can lead to rapid multisystemic complications, including severe interstitial lung disease (ILD). This case, involving a 60‐year‐old female with worsening skin lesions and ILD, demonstrates the disease's aggressive nature, highlighting the need for early diagnosis and comprehensive immunosuppressive therapy to improve patient outcomes.

## 1 Introduction

Among the several forms of dermatomyositis (DM), clinically amyopathic dermatomyositis (CADM) is a rare condition with an annual incidence of 2.08 per million people [1] and comprises about 20% of the total DM population. CADM refers to the group of patients with biopsy‐proven cutaneous findings of DM that never manifest clinical weakness or markedly elevated muscle enzymes but may have subclinical muscle abnormalities on electromyogram, MRI, or muscle biopsy studies. MDA‐5 antibody is strongly associated with ILD (90%–95%) in CADM, with a particularly high risk of rapidly progressive ILD (RP‐ILD), which occurs in up to 80% of cases [2, 3]. MDA‐5 have a prevalence of 0%–13% in caucasians, 11%–57% in Asians, and 7%–12% in juvenile DM. Limited understanding exists regarding the pathogenic mechanisms of anti‐MDA5 DM due to its rarity, although it is thought to result from specific gene–environment interactions, with documented HLA allele associations in Asian populations [4]. Gottron's sign and papules are the pathognomonic cutaneous lesions associated with DM [5]. Early administration of a treatment regimen combining high doses of systemic glucocorticoids with other immunosuppressive medications like calcineurin inhibitors and/or cyclophosphamide seems to offer the best chances of survival for individuals with CADM associated with RP‐ILD, whereas for refractory disease, further treatments like plasma exchange can be introduced [6, 7].

## 2 Case Report

**FIGURE 1:**
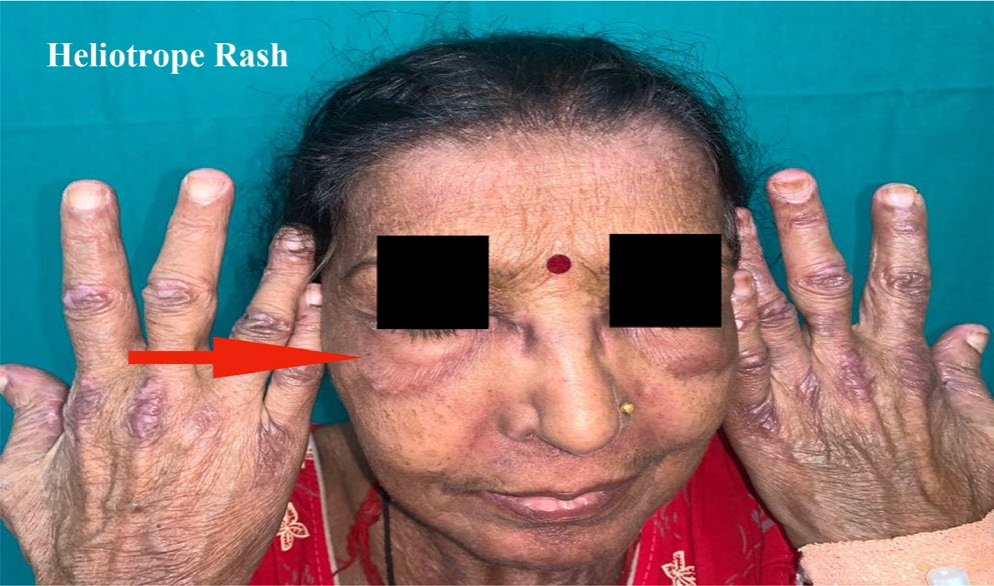
Heliotrope rash—Violaceous erythema involving periorbital region.

**FIGURE 2:**
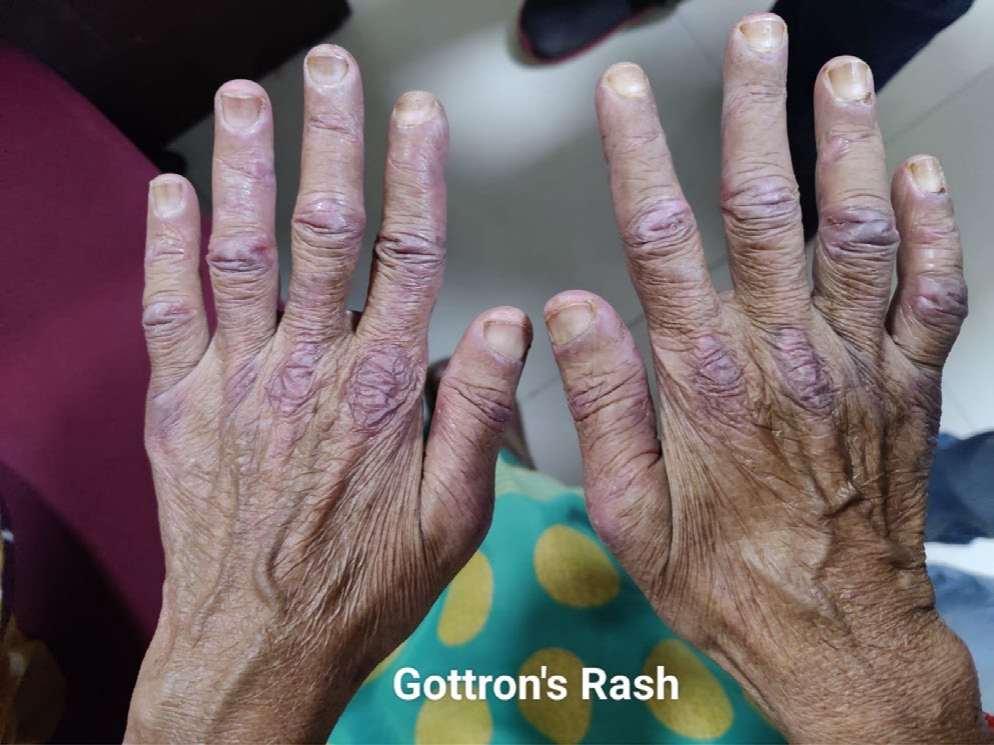
Gottron's rash—Plaques/Papules over MCP and PIP joints.

**FIGURE 3:**
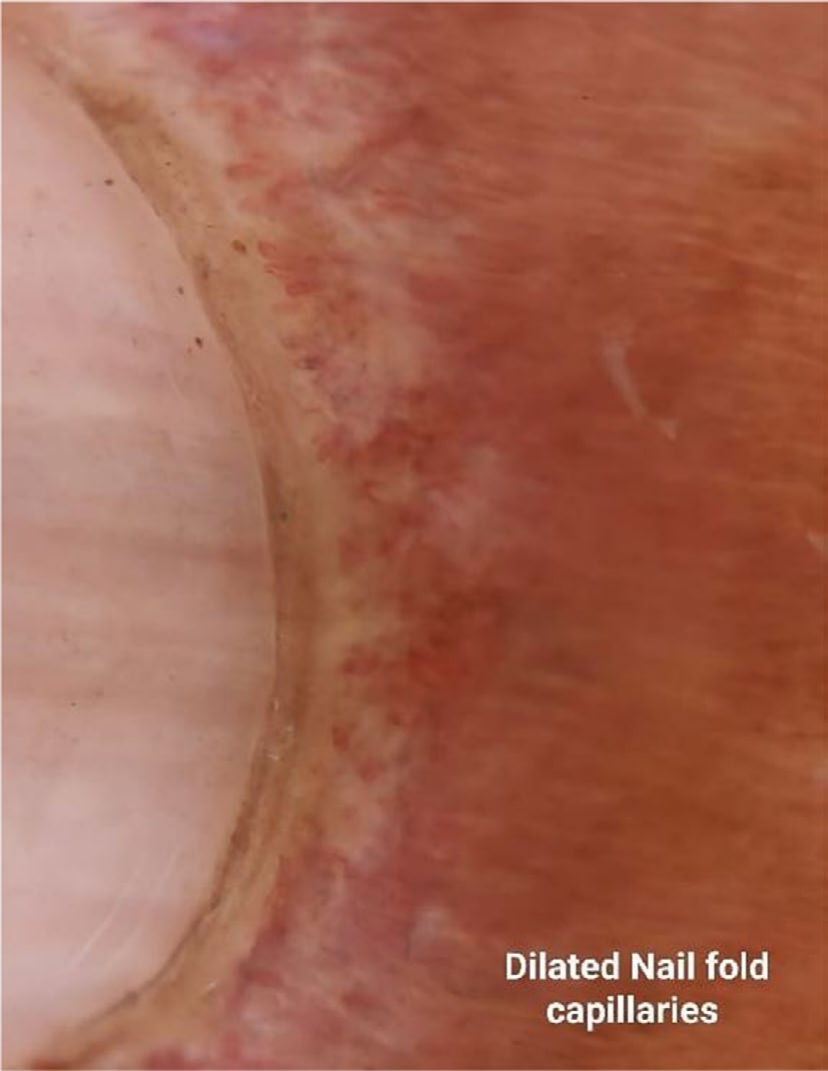
Nail fold telangiectasia‐Dilated nail fold capillaries.

**FIGURE 4:**
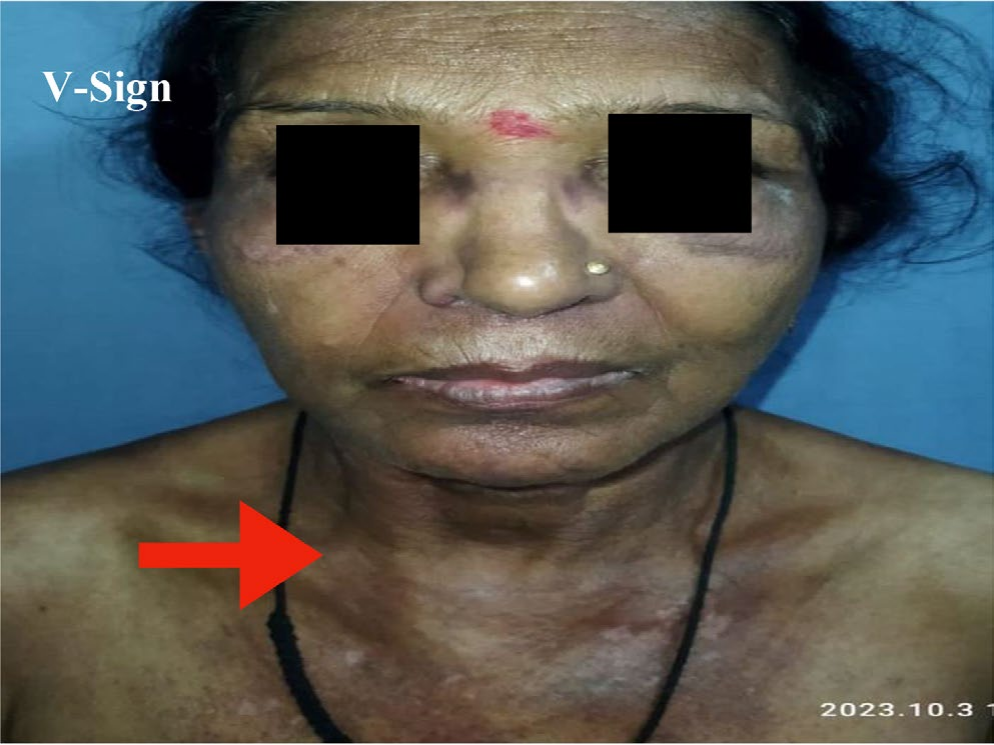
V‐sign—Violaceous erythema involving front of neck.

**FIGURE 5:**
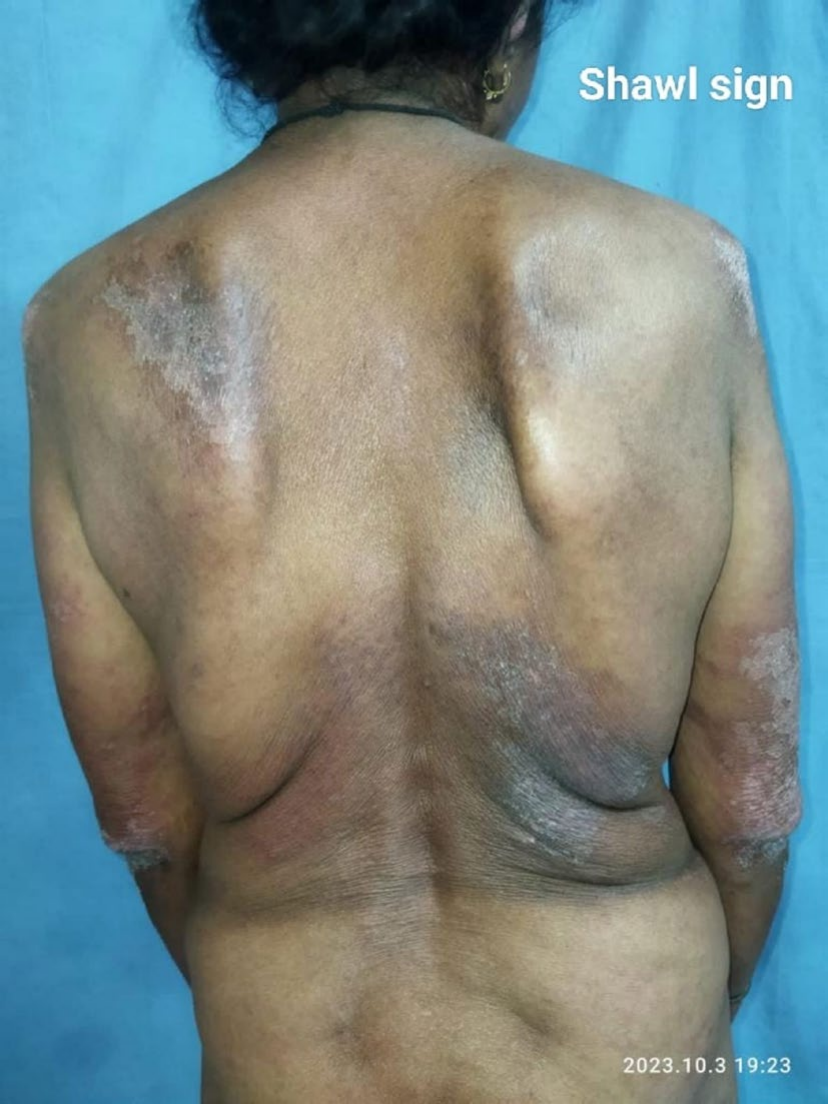
Shawl sign‐Erythema involving back region.

A 60‐year‐old female patient presented with complaints of dry cough for 2 months, breathlessness on exertion for the last 15 days, and fever for 5 days. There was no complaint of exposure to allergens, weight loss, dyspepsia, pedal edema, orthopnea, chest pain, decreased urine output, muscle weakness, or joint pain. The patient had a past history of multiple skin lesions over the hands, face, chest, shoulder, and gluteal region for the last 6 months, and oral ulcers for 3 months, for which she received medications, but her symptoms continued to worsen. On general examination, the respiratory rate was 26/min and oxygen saturation was 94% (for which she was given nasal O2), while other vitals were stable. On head to toe examination, a violaceous erythematous rash (Heliotrope rash [Figure 1]) involving the periorbital region, Gottron papules (Figure 2) over the metacarpophalangeal and proximal interphalangeal joints, dilated nail fold capillaries (Figure 3) plaques over the extensor surface of elbow (Gottron sign) and erythema over the front of neck (Figure 4) and back (Figure 5) were observed, which strongly suggested the clinical finding of DM.

Despite repeated inquiry, the patient denied having muscle pain or weakness ever, and there was no objective evidence of decreased muscle power on CNS examination. On Respiratory system examination, tactile vocal fremitus was increased in the left infra scapular area. Also, fine Velcro crepitations were heard bilaterally in the infra scapular area (ISA), Infra axillary area (IAA) and Inframammary area (IMA) with bronchophony in the left ISA region on auscultation. A dull note over the left IAA and ISA region was percussed. Laboratory examinations were as follows:

Has elevated ESR suggestive of inflammatory changes, also has mild anemia; elevated CPK indicates muscle damage despite the absence of symptoms. (Table 1).

**TABLE 1:** WBC‐White Blood Cells ESR‐Erythrocyte Sedimentation Rate, T3‐Triiodothyronine, T4‐Thyroxine, TSH‐Thyroid Stimulating Hormone, TPO‐Thyroid peroxidase.

Parameter	Value	Reference range
Hemoglobin	10.34 g/dL	11–15 g/dL
Total WBC count	10,900/cumm	4000–11,000/cumm
Platelet count	158,000/cumm	1.5–4.1 L/cumm
ESR	50 mm	0–19 mm
Biochemistry		
Urea	19 mg/dL	14–40 mg/dL
Creatinine	0.7 mg/dL	0.6–1.2 mg/dL
Total bilirubin	0.8 mg/dL	0.1–1.2 mg/dL
Total Creatinine Phosphokinase (CPK)	464 IU/L	0–145 IU/L
Thyroid Profile		
Free T3	1.69 pg/mL	2–4.4 pg/mL
Free T4	1.32 ng/mL	0.93–1.7 ng/mL
TSH	22.99 μIU/ml	0.27–4.2 μIU/ml
Anti‐ TPO antibody	< 5 IU/mL	0–35 IU/mL

**FIGURE 6:**
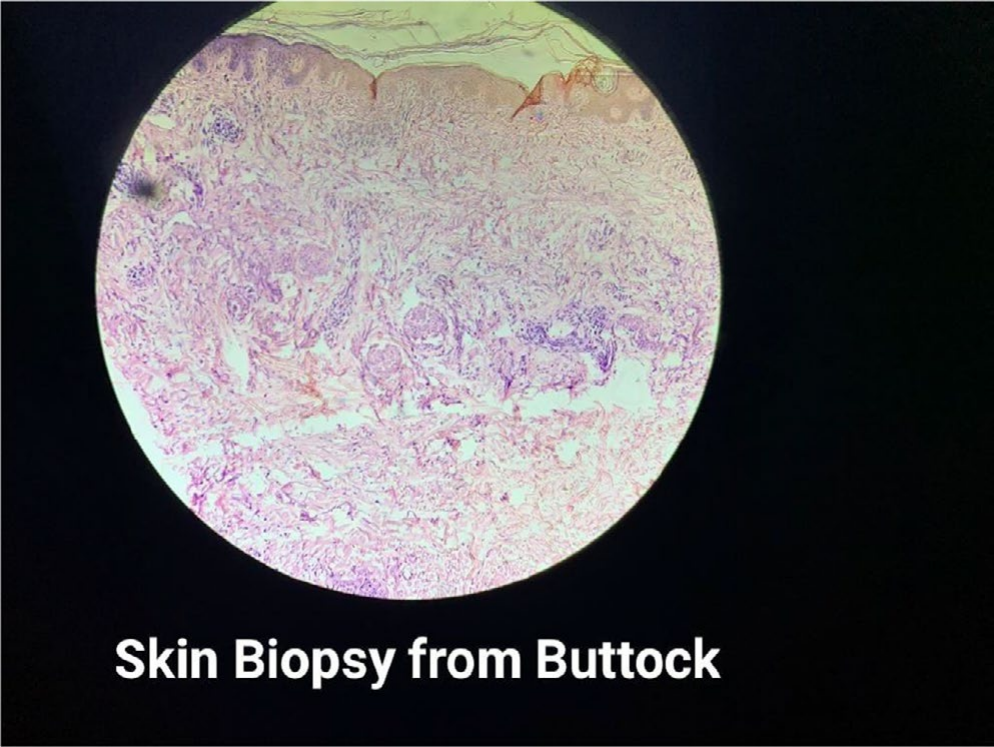
Skin Biopsy. Shows focal thinning of epidermis with subtle vacuolar changes. Underlying dermis shows aggregates of lymphocytes, histiocytes and few plasma cells with areas of hemorrhage. S/O—DERMATOMYOSITIS.

**FIGURE 7:**
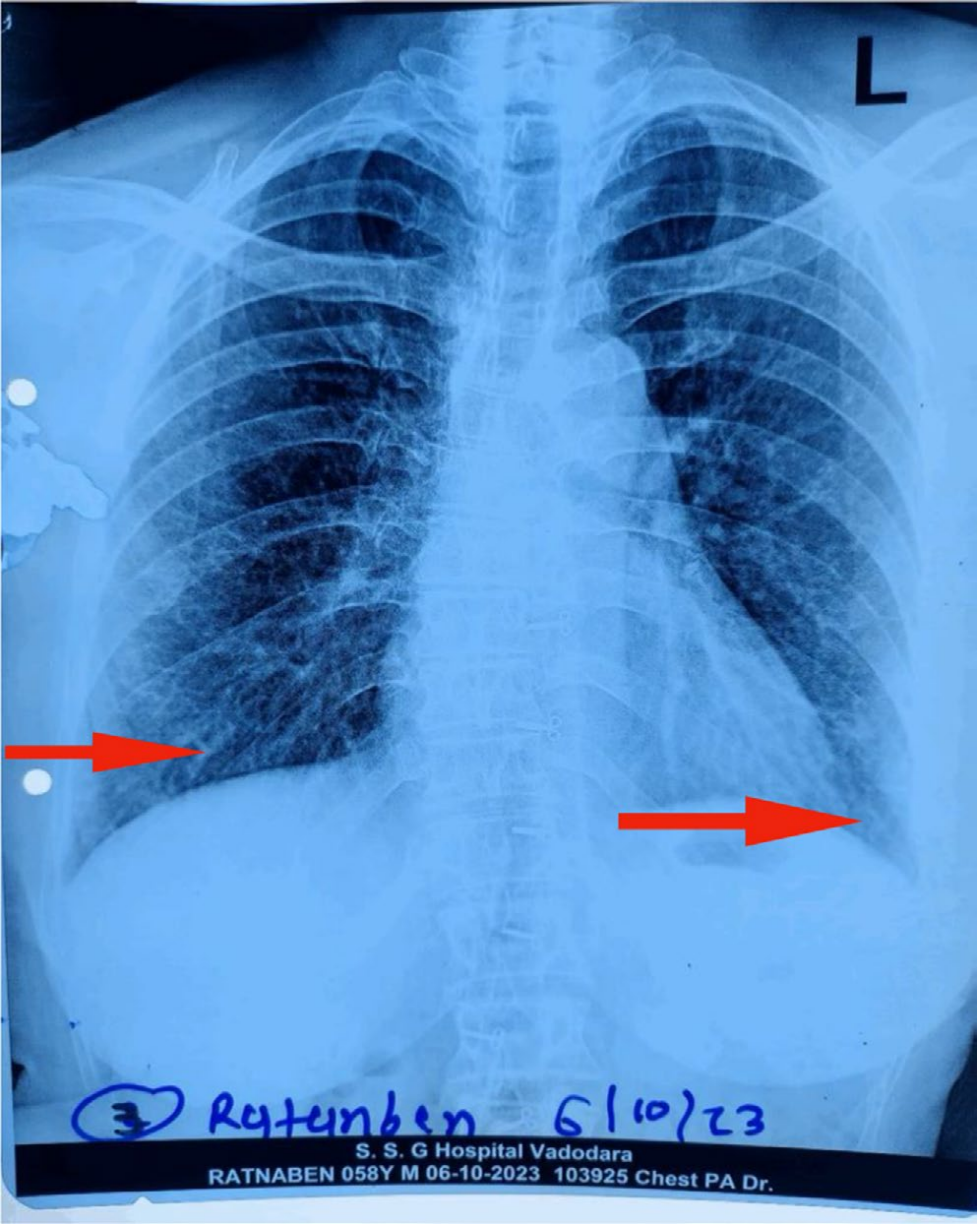
Reticular opacities noted in B/L lower and left middle lung zones.

**FIGURE 8:**
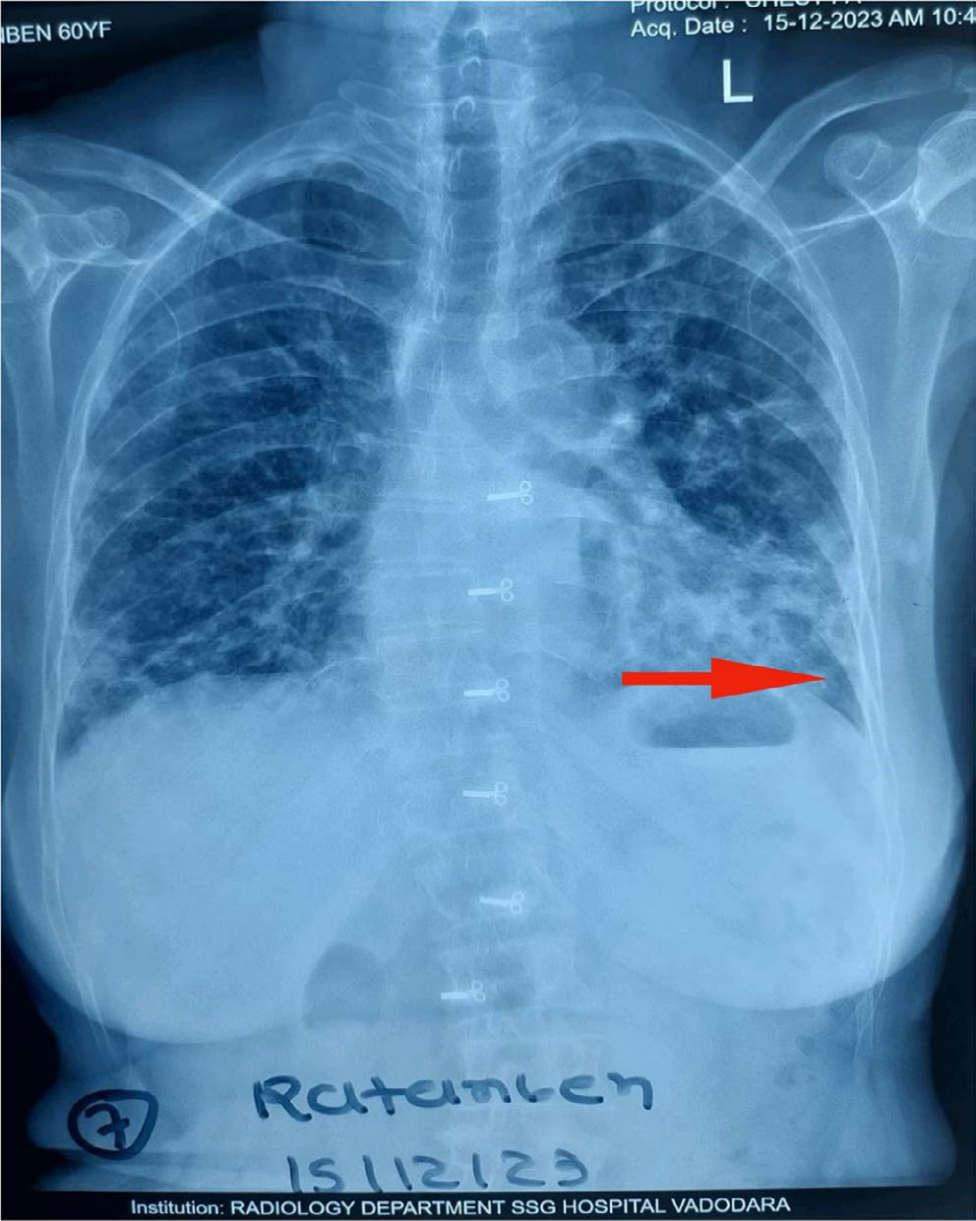
Radio‐opacity noted in left lower lung zone‐p/o consolidation.

**FIGURE 9:**
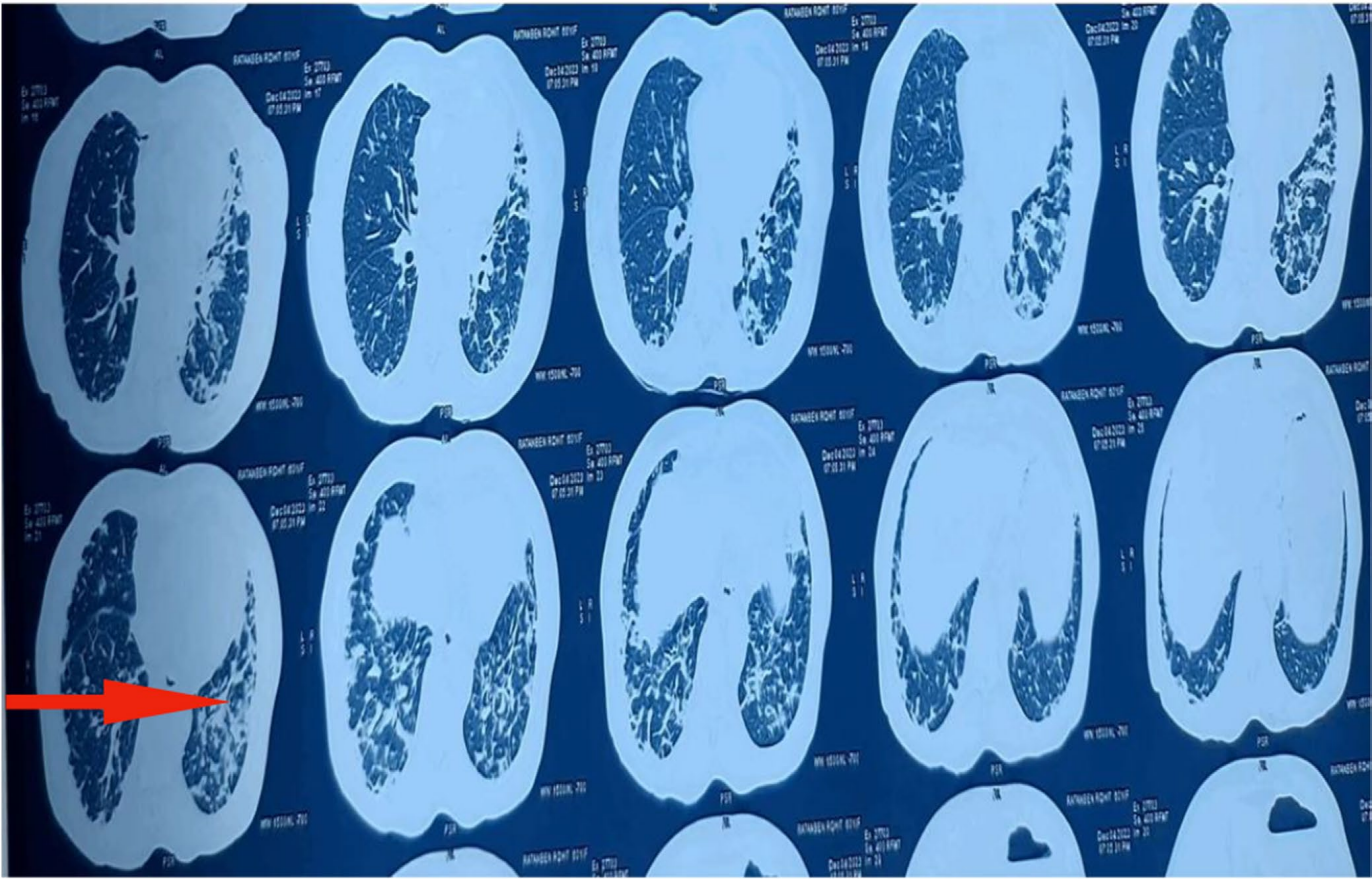
HRCT Chest. Multifocal diffusely scattered ground glass and reticular densities and consolidation in both the lungs predominantly on left side represent infective/inflammatory etiology. Few subcentimeter size bilateral hilar, pre and paratracheal, paraaortic, aorto‐pulmonary window and subcarinal lymphnodes. Dilated right and left pulmonary arteries may represent pulmonary arterial hypertension.

Skin biopsy (Figure 6) showed focal thinning of the epidermis with subtle vacuolar changes, and the underlying dermis showed aggregates of lymphocytes, histiocytes, and a few plasma cells with areas of hemorrhage. T inversion in V1‐V5, left axis deviation, and sinus tachycardia were present on the Electrocardiogram. Reticular opacities were noted in the bilateral lower and left middle lung zones, and radio‐opacity was noted in the left lower lung zone on the Chest X‐Ray (Figures 7 and 8). HRCT Chest (Figure 9) showed Diffuse ground‐glass opacities and consolidations in both lungs, predominantly left‐sided, suggestive of an infective/inflammatory cause. Additionally, small bilateral hilar and mediastinal lymph nodes were noted, alongside dilated pulmonary arteries indicative of possible pulmonary arterial hypertension. On the ANA and myositis profile, Ro‐52 and MDA 5 were found to be strongly positive, with fine speckled immunofluorescence and homogeneously positive cytoplasm giving the impression of CADM with Rapidly progressive Interstitial Lung Disease in a case of Hypothyroidism.

## 3 Differential Diagnosis

While the patient presented with skin conditions and positive ANA, similar to other autoimmune diseases, the absence of specific symptoms (like joint pain, dry mouth, etc.) and the presence of anti‐MDA‐5 antibodies strongly suggest a diagnosis of anti‐MDA‐5 positive amyopathic DM.

Other potential diagnoses, such as SLE, Sjögren's Syndrome, and idiopathic interstitial pneumonia with autoimmune features (IPAF), were considered but ruled out due to the lack of specific symptoms or the presence of conflicting markers. Conditions like mixed connective tissue disease (MCTD), Systemic Sclerosis, and Paraneoplastic DM were also considered but were less likely given the patient's symptoms and test results.

Drug‐induced myopathy and viral myositis were unlikely due to the absence of a relevant medication history and the chronic nature of the patient's symptoms. Psoriasis was also considered but was ruled out based on the lack of typical psoriatic features.

Considering the overall clinical picture and the presence of anti‐MDA‐5 antibodies, the most likely diagnosis is anti‐MDA‐5‐positive amyopathic DM, with a focus on early treatment due to the associated risk of rapid disease progression.

## 4 Treatment

The patient was given Injection Ceftriaxone 1 g intravenously every 12 h for 5 days: This broad‐spectrum antibiotic is likely prescribed to treat or prevent secondary bacterial infections, which are common in immunocompromised patients or those on high‐dose steroids. Tablet Thyroxine 50 micrograms once a day before breakfast: This addresses hypothyroidism, which could be a coexisting condition. Thyroid hormone replacement ensures optimal metabolism and supports the patient's general health. Tablet Prednisolone 5 mg, once a day with milk for 1 week: 2 This corticosteroid is a cornerstone treatment for inflammation and immune suppression in CADM. The dose increase reflects disease progression or inadequate response to prior therapy. Milk is recommended to reduce gastrointestinal side effects. Tablet Mycophenolate Mofetil 360 mg twice a day for week: An immunosuppressant that helps control autoimmunity and prevents further damage to lung tissue and other organs. Injection Rituximab (one dose): A monoclonal antibody targeting B cells, given to suppress the immune response further and treat RP‐ILD, especially in anti‐MDA5‐positive patients. Injection Cyclophosphamide 750 mg in 500 cc Normal Saline over 4–5 h: A potent cytotoxic and immunosuppressive agent used to manage severe RP‐ILD. The administration of MESNA following cyclophosphamide helps prevent bladder toxicity. Tablet Calcium 500 mg twice a day and Vitamin D3 sachet once a week: These are supportive therapies to counteract steroid‐induced osteoporosis, ensuring bone health during long‐term corticosteroid use. Syrup containing Phenylephrine, Chlorpheniramine Maleate, and Dextromethorphan Hydrobromide (DMR), 2 tablespoons thrice a day: This combination syrup addresses upper respiratory symptoms such as congestion, allergy, and cough, possibly linked to respiratory tract involvement or coexisting infection. Tablet Tacrolimus 0.5 mg once daily (starting after week): This calcineurin inhibitor modulates T‐cell activity, complementing other immunosuppressants in controlling autoimmunity, particularly in lung involvement. This regimen reflects a comprehensive approach targeting both the CADM with RP‐ILD and its complications, with careful consideration of supportive care to mitigate treatment side effects. Frequent monitoring of organ function (e.g., liver, kidneys, and lungs) and drug side effects was done during such an intensive therapy course. The patient was improving at a slow rate and wanted discharge after a week and was given in accordance.

## 5 Follow‐Up and Outcome

Patient was planned for weekly follow‐up till 1 month and then on biweekly follow‐up for the next 3 months. Patient came directly after 1 month; the patient's condition was not improving despite treatment and was suggested for inpatient care for further management, but the patient had taken discharge and was prescribed medication as asked by the patient. After 10 days of discharge, she came back in as her condition deteriorated at home and she was not able to breathe, and had fainted, and was brought to the hospital where she was admitted to the intensive care unit (ICU) due to ILD progressing rapidly despite treatment. On admission that day, she had very low saturation (SpO2‐55%) also had Total Creatinine Phosphokinase (CPK) very elevated (1450 IU/L) and Chest X‐ray findings were rapidly progressive interstitial lung disease (RP‐ILD) including bilateral reticulonodular patterns, ground‐glass opacities, and honeycombing. Changes were basilar and showed superimposed consolidation more on the left side. There was worsening shortness of breath, hypoxia, and cough despite treatment, and died on the 4th day of her intensive care admission.

## 6 Discussion

Dermatomyositis is a type of idiopathic inflammatory myopathy (IIM) characterized by an inflammatory infiltrate that primarily targets the skeletal muscles and skin, often accompanied by distinctive skin lesions. Several subsets of DM have been identified to date. These include: classic DM, where both muscle and skin are affected; CADM, which involves skin but not muscle; hypomyopathic DM, characterized by skin symptoms combined with subclinical signs of myositis; postmyopathic DM, observed in patients with prior classic DM who recover from myositis but continue to experience active skin rashes; and DM sine dermatitis, where no skin rash is present, but muscle biopsy findings are indicative of DM [8].

Clinically Amyopathic Dermatomyositis primarily affects young adults; also, cases of juvenile onset have been reported in the literature [9]. Previous studies have shown that patients with DM who are positive for anti‐Tif1γ antibodies face a higher risk of cancer‐related complications. Conversely, anti‐MDA‐5 antibodies have not been commonly linked to an elevated cancer risk [10, 11]. The literature contains limited reports of cancer in patients with anti‐MDA‐5 antibody‐positive rapidly progressive interstitial lung disease (RP‐ILD) [12, 13].

CADM primarily affects the skin but can also impact various organ systems like pulmonary –IDL (interstitial lung disease), rheumatology‐Arthralgia, myositis and Arthritis, GI– dysphagia, cardiac –rdiomyopathy, etc. Muscle inflammation in myositis is associated with higher amounts of molecules mistaken by the body's defense system as harmful. This connection suggests a link between cancer and this muscle disease because these molecules are found in both cancerous and muscle tissues. The immune responses directed at tumors may also cause damage to muscle tissue in cases of DM or polymyositis (PM) [14]. Recent studies have revealed a paraneoplastic association between DM and cancer, with approximately 24% of DM cases linked to malignancies [15]. These observations have the important role of immune dysregulation in the etiology of DM and its significance as an indicator of concealed malignancies. Interestingly, the patient initially presented with suspected psoriasis. Psoriatic lesions can resemble Gottron's papules, and therefore, especially in the absence of muscular symptoms, may be misdiagnosed [7].

Amyopathic dermatomyositis (ADM) and hypo‐myopathic dermatomyositis (HDM) are subtypes of dermatomyositis distinguished by the degree of muscle involvement. ADM is characterized by the presence of classic skin manifestations, such as Gottron's papules and a heliotrope rash, without any clinical or laboratory evidence of muscle involvement. In ADM, muscle enzymes like creatine kinase (CK) and aldolase remain normal, and there is no muscle weakness or inflammation detected through electromyography (EMG) or biopsy. On the other hand, HDM also presents with the characteristic skin findings of DM but includes subclinical muscle involvement. In HDM, mild abnormalities may be detected in muscle enzymes, EMG, or biopsy, even though the patient does not exhibit significant muscle weakness. In summary, the primary distinction is that ADM lacks any muscle involvement, while HDM involves mild, subclinical abnormalities without overt muscle weakness.

This case underscores the intricate interaction of various systemic and autoimmune disorders, culminating in a fatal outcome. The patient, a 60‐year‐old woman, presented with deep, painful skin ulcers and palmar papules, also known as inverse Gottron's papules, which are more characteristic of anti‐MDA‐5 DM. Histological analysis of DM skin usually shows interface dermatitis. In the MDA‐5 subtype, classic symptoms of DM, including epidermal necrosis and vasculopathy, are observed, often accompanied by rapidly progressive interstitial lung disease (RP‐ILD) and hypothyroidism [16]. The patient was administered a comprehensive treatment plan, which included antibiotics (Ceftriaxone) to address potential infections, immunosuppressive agents (Prednisolone, Mycophenolate Mofetil, Rituximab, Cyclophosphamide), and supportive therapies (Thyroxine, Calcium, Vitamin D3, and antitussive syrup). The intensification of immunosuppressive therapy, particularly with Rituximab and Cyclophosphamide, highlighted the critical and rapidly worsening condition of the ILD. For patients with resistant DM affecting the lungs and esophagus, intravenous immunoglobulin (IVIG) is crucial in treatment [17]. Studies have demonstrated that IVIG reduces complement activity, limits the deposition of the membrane attack complex on capillaries and muscle fibers, and decreases the expression of adhesion molecules and cytokine production [18, 19].

This case highlights the complex diagnostic and therapeutic challenges involved in managing such intricate presentations. Despite aggressive treatments, the patient's condition worsened, resulting in death.

Perform baseline and periodic PFTs, including DLCO, to detect ILD early. Use HRCT for subclinical ILD in CADM. Check myositis‐specific autoantibodies (anti‐MDA5, anti‐TIF1‐γ, and anti‐NXP2), inflammatory markers (ESR, CRP, and ferritin), imaging, endoscopy, and tumor markers. Monitor CK and aldolase levels, even without muscle symptoms, to identify subclinical myositis and assess complication risks [20].

“When a prevention campaign works, nothing happens!” ~Norman Swan.

Thus, for patients with anti‐MDA‐5 antibody‐positive CADM, early screening and consistent monitoring of complications are crucial to enhance morbidity outcomes and reduce mortality rates.

## Author Contributions

**Hem Prajapati:** conceptualization, resources, writing – original draft, writing – review and editing. **Yesha R. Chauhan:** resources, validation, writing – original draft. **Sahaj Y. Patel:** writing – original draft, writing – review and editing. **Ajay C. Parmar:** resources, supervision, validation. **Tasin Mohammedyakub Shaikhjiwala:** resources, writing – review and editing.

## Consent

We have taken written consent from the patient regarding publishing clinical information as well as radiological image findings of the patient, which would be open access, and the patient has agreed to this condition.

## Data Availability

The data that support the findings of this study are openly available in Authoria at https://doi.org/10.22541/au.172526600.03613776/v1.
